# Effect of Laser-Exposed Volume and Irradiation Position on Nonphotochemical Laser-Induced Nucleation of Potassium Chloride Solutions

**DOI:** 10.1021/acs.cgd.3c00865

**Published:** 2023-10-16

**Authors:** Vikram Korede, Mias Veldhuis, Frederico Marques Penha, Nagaraj Nagalingam, PingPing Cui, Antoine E.D.M. Van der Heijden, Herman J.M. Kramer, Hüseyin Burak Eral

**Affiliations:** † Process & Energy Department, Delft University of Technology, Leeghwaterstraat 39, 2628 CB Delft, The Netherlands; ‡ Department of Chemical Engineering, KTH Royal Institute of Technology, Teknikringen 42, 114 28 Stockholm, Sweden; ¶ School of Chemical Engineering and Technology, State Key Laboratory of Chemical Engineering, Tianjin University, Tianjin 300072, People’s Republic of China

## Abstract

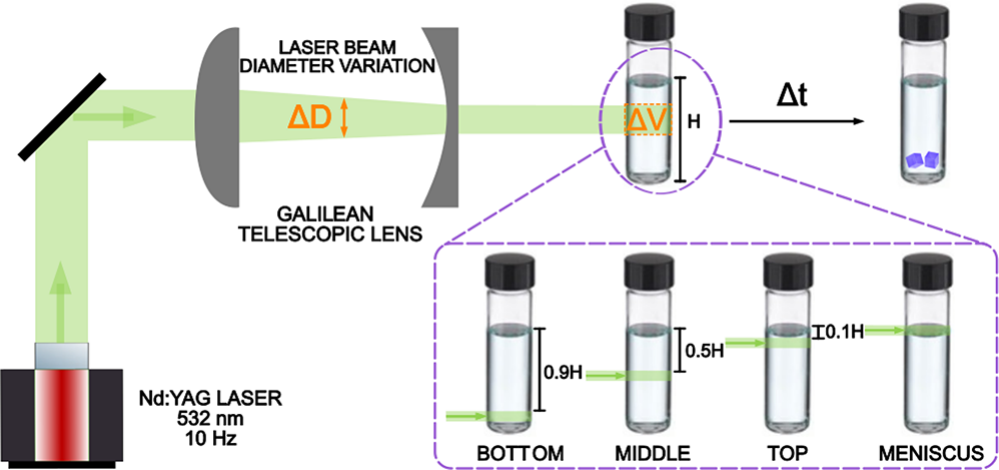

Herein, we study the influences of the laser-exposed volume and the irradiation position on the nonphotochemical laser-induced nucleation (NPLIN) of supersaturated potassium chloride solutions in water. The effect of the exposed volume on the NPLIN probability was studied by exposing distinct milliliter-scale volumes of aqueous potassium chloride solutions stored in vials at two different supersaturations (1.034 and 1.050) and laser intensities (10 and 23 MW/cm^2^). Higher NPLIN probabilities were observed with increasing laser-exposed volume as well as with increasing supersaturation and laser intensity. The measured NPLIN probabilities at different exposed volumes are questioned in the context of the dielectric polarization mechanism and classical nucleation theory. No significant change in the NPLIN probability was observed when samples were irradiated at the bottom, top, or middle of the vial. However, a significant increase in the nucleation probability was observed upon irradiation through the solution meniscus. We discuss these results in terms of mechanisms proposed for NPLIN.

## Abstract 2

The nonphotochemical laser-induced nucleation (NPLIN) probability of KCl solutions was systematically investigated under varying conditions, including different laser-exposed volumes (by changing the laser beam diameters) and irradiation positions. These experimental findings were evaluated in the context of NPLIN mechanisms previously proposed in the literature. The results could pave the way for industrial-scale applications of NPLIN.

## 1 Introduction

Controlling nucleation in a crystallization process has direct implications on the production of crystals that we encounter in products ranging from pharmaceuticals to explosives.^1−6^ Alternative crystallization methods, including nonphotochemical laser-induced nucleation (NPLIN), have been extensively studied over the past decades to provide spatiotemporal control over crystal nucleation. In NPLIN, a supersaturated solution is exposed to an intense laser pulse that induces nucleation in a drastically reduced induction time without absorbing any light at the irradiated wavelength.^5,7^

Ever since its discovery, NPLIN has been observed in many systems: small inorganic compounds,^8−10^ small to large organics,^11−16^ proteins,^17,18^ liquid crystals,^19^ supercooled liquids,^10^ and even gas bubbles.^20,21^ Subsequently, the NPLIN effect with key experimental observations can be summarized as the following: (i) nucleation probabilities appear to be independent of the tested laser wavelengths and are a function of supersaturation and the peak intensity of the laser;^13,17,22,23^ (ii) there exists a threshold peak intensity below which nucleation is not induced;^8,10,13,23,24^ (iii) while not a requirement for NPLIN, the aging of samples has been shown to have an influence on the nucleation probabilities upon laser irradiation;^11,23,25^ and (iv) the presence of added nanoparticles that act as impurities enhances the nucleation probability of NPLIN.^26,27^ These observations are summarized and discussed at length in a recent review article.^5,7^

Several mechanisms have been proposed to explain the aforementioned experimental observations. The first proposed mechanism is based on the optical Kerr effect (OKE), where molecules in precritical clusters may align with the direction of the laser electric field, thus facilitating nucleation.^28^ This mechanism corroborates observations that some polymorphs could be favored depending on the laser characteristics (intensity, wavelength, and polarization). However, Monte Carlo simulations of a Potts lattice gas model cast doubt on the amount of energy needed to properly align molecules in precritical solute clusters, as the field strength needed to lower the nucleation barrier to match the NPLIN observations was found to be orders of magnitude higher than the field strengths employed in the experiments.^21^ In addition, later studies on aqueous potassium chloride systems showed that NPLIN of small inorganic compounds without a preferential polarization axis was also possible, ruling out the OKE mechanism for KCl.^23^

Alexander et al.^23^ proposed the dielectric polarization (DP) model to quantitatively describe the influence of a laser beam incident on aqueous potassium chloride solutions. The model exploits the difference between the dielectric constant of a cluster of solute molecules, ϵ_p_, and the dielectric constant of the surrounding medium, ϵ_S_. In the presence of an electric field and under the constraint that ϵ_p_ > ϵ_S_, the free energy change of cluster formation (*ΔG*) is lowered by a number proportional to −*v*(ϵ_p_ – ϵ_S_)*E*^2^, where *v* is the volume of the cluster and *E* is the electric field strength.^5^ In the presence of an electric field, the critical radius, *r*_c_(*I*), and the height of the nucleation barrier, *ΔG*_c_(*I*), are calculated using the classical nucleation theory approach. As a result of the decreased nucleation barrier, any existing precritical clusters become critical following exposure to the laser, thus inducing nucleation. According to Alexander et al., the number of precritical clusters needed to become viable crystals (*N*_crystals_) is a function of the lability constant, *m*, and the peak laser intensity, *I*: *N*_crystals_ = *mI*, where *m* is a function of the solute/solvent characteristics and the number of solute molecules present in the volume irradiated by the laser. However, studies investigating the NPLIN effect on carbon dioxide bubbles showed that NPLIN is also possible when ϵ_p_ > ϵ_S_ is not satisfied,^20,29^ casting doubt on the applicability of the DP model.

The third potential mechanism that has been proposed is based on the heating of nanoparticle impurities inherently present in the system, such as soluble molecular impurities (intrinsic) and/or dust particles (extrinsic), when they are exposed to the laser beam. The heating of these nanoparticles results in the vaporization of a volume of the liquid surrounding them. This leads to the formation of a vapor cavity, following which a region of increased solute concentration may form near the vapor/liquid interface, upon which the solute molecules are more likely to cluster and nucleate. A consensus on which model accounts for all of the observations has still not been reached. While the optical Kerr effect and the DP model can explain several observations in the NPLIN experiments, they fail to explain the nucleation of carbon dioxide bubbles, the decrease in the nucleation probability by the filtration of the solution, or the increase in the nucleation probability by intentionally doping the system with nanoparticles.^20,21,26,27^

Among the many studies published in the NPLIN literature, various parameters, including the supersaturation, laser intensity, polarization,^8,10,13,14,30^ sample filtration,^26^ and intentional doping with impurities,^27^ have been shown to influence the measured nucleation probabilities. Despite the broad literature on NPLIN, the comparison of different reports is challenging due to variations in the many crucial experimental parameters, such as the geometry (e.g., container geometry, impurity content, and how the beam interacts with the confining surfaces and solution), laser characteristics (e.g., intensity, wavelength, polarization, type of laser (continuous or pulsed), and pulse width), time scale of exposure (ranging from femtoseconds in the case of a single pulse to series of pulses repeatedly exposing solutions for as long as 1 h),^27^ and solution characteristics.

**Figure 1:**
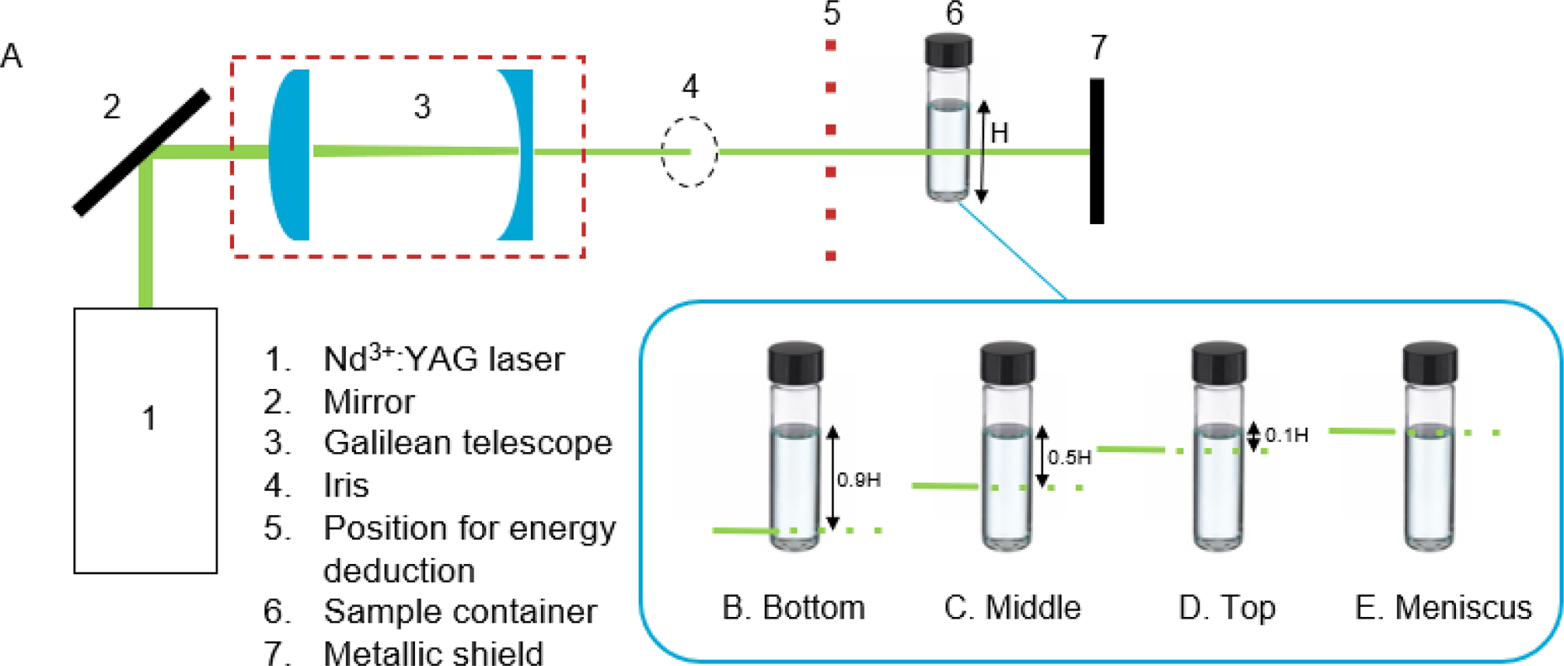
(A) The experimental setup used throughout the laser-exposed volume experiments. The exposed volume was controlled by a Galilean telescope. The height of liquid in the vial from the air/solution interface to the bottom of the vial is denoted as H. (B–E) The different irradiation locations and the location of the exposed volume with respect to the air/solution interface at a fixed laser intensity and supersaturation. The laser beam is illustrated as a green line.

Interestingly, the effect of the amount of solution volume exposed to the laser beam, a critical experimental parameter for the industrial implementation of NPLIN, has been previously explored to a certain extent. Fang et al. reported a significant volume dependence when comparing the nucleation rate of aqueous KCl in a test tube to that in a levitated microdroplet, where the ratio of irradiated volumes was as dramatic as 40 million times.^31^ In addition, a study by Hua et al.^32^ provided insights into the microfluidic laser-induced nucleation of supersaturated KCl solutions. This work outlined a continuous flow system for NPLIN, in which each volume element of the solution was subjected to laser pulses. The system allowed for a varied irradiated volume by changing the flow time, and it was shown that the number of crystals that formed was directly proportional to the laser intensity. In light of these studies, our current investigation builds on the existing knowledge in order to provide a comprehensive understanding of how varying the laser-exposed volume impacts the nucleation probability in milliliter-scale volumes of supersaturated aqueous potassium chloride. The results were then analyzed with the dielectric polarization mechanism and classical nucleation theory. The effect of supersaturation and intensity was also studied to determine their influence on the laser-exposed volume dependency. Furthermore, the position of laser irradiation with respect to the air/solution interface (e.g., near the bottom of a cylindrical vial, in the bulk of the solution, near the air/liquid interface, and directly at the air/solution interface, as illustrated in Figure 1) was studied to quantify how laser positioning alters the nucleation probability.

## 2 Experimental Section

### 2.1 Laser-Exposed Volume Experiments

#### 2.1.1 Solution Preparation

Stock solutions were prepared by adding weighed amounts of ultrapure water (Elga PURELAB, U.K., 18.2 MΩ cm) to potassium chloride (Sigma-Aldrich, molecular biology grade, ≥99.0% purity, CAS: 7447-40-7) in flasks corresponding to concentrations of 0.370 and 0.375 g_KCl_/g_H_2_O_, which yielded solutions with supersaturations of 1.034 and 1.050 at a temperature of 24 °C, respectively. The supersaturated solutions were placed in an oven at 50 °C overnight to ensure the complete dissolution of the potassium chloride crystals. The solution flasks were then transferred to a hot plate and stirred (at 50 °C and 400 rpm) before the solutions were distributed across 100 8 mL borosilicate high-performance liquid chromatography (HPLC) vials (BGB, dimensions of 61 × 16.6 mm). Each vial was filled with 7 mL of solution using a bottle-top dispenser. All of the sample vials were stored in an oven operating at 50 °C for at least one night before use. Before the laser experiments were conducted, the sample vials were transferred from the oven to a thermostatic bath (Lauda Eco R E620) operating at 50 °C and then subsequently cooled overnight to 24 °C. Furthermore, for every experiment, the samples were aged in a thermostatic bath set to 24 °C for a duration of 6 h. It should be noted that the 6 h aging period commenced only once the bath temperature stabilized at 24 °C, and this duration did not include any cooling time from higher temperatures.

#### 2.1.2 Laser Setup and Sample Handling

A schematic of the setup used in the laser-exposed volume experiments is shown in Figure 1A. A Q-switched Nd:YAG laser (Continuum, Powerlite DLS 8000) was used to generate 7 ns pulses of linearly polarized light at a wavelength of 532 nm and a frequency of 10 Hz. The direction of the generated light was changed by the use of a mirror (NB1-K13, Thorlabs) or a beamsplitter (BSN10, Thorlabs), depending on the intensity requirements of the laser. The fundamental beam, which was 9 mm in diameter, was then passed through a Galilean telescope with lens configurations of different focal lengths to either reduce or increase the laser beam diameter in order to vary the laser-exposed volume. An iris that was adjusted to a slightly larger size than the beam diameter served as a filter for any artifacts produced by the laser. An overview of the mirrors and lenses used and the resulting beam diameters is given in Table 1.

**Table 1:** Mirrors and Lens Configurations Used in the Laser-Exposed Volume Experiments^a^

mirror reflectance (%)	focal length plano-convex *f*_a_ (mm)	focal length plano-concave *f*_b_ (mm)	resulting beam diameter (mm)	resulting laser-exposed volume^a^*V*_laser_ (cm^3^)
10	200	–50	2.3	0.047
10 or 100	150	–75	4.5	0.179
100	N/A	N/A	9.0	0.705
100	100	–75	12.0^b^	1.230
100	150	–100	13.5^b^	1.538

a Laser-exposed volume of the vials containing aqueous potassium chloride solutions with a supersaturation of *S* = 1.034 at 24 °C.

b Position of the lenses interchanged.

Prior to irradiation of the samples, the average pulse energy of the laser beam was recorded by taking the average of 20 pulses using an energy meter (QE25LP-H-MB-QED-D0, Gentec-EO). For experiments focused on the effect of the exposed volume, the position of the light incident on the sample vial was chosen to be in the middle with respect to the bottom of the vial and the meniscus of the solution.

Care was taken to not induce nucleation by any mechanical shock and to keep the vials vertical at all times while the solutions were transferred from the thermostatic bath to the setup during the experiment. One by one, the samples were moved from the bath, carefully dried with a fabric cloth, and subsequently checked for crystal formation. If at this point crystals were observed, the sample was omitted from the data set. If not, the vial was exposed to a single laser pulse by varying the laser beam diameter (2.3, 4.5, 9, 12, and 13.5 mm) at different laser intensities (10 and 23 MW/cm^2^). After laser irradiation, the sample was immediately moved back to the bath operating at 24 °C. The vials were checked for crystals after 80 min. The observation time of 80 min was chosen by considering previous literature,^8^ where a detection time of 60 min has been reported to be sufficient for crystals to be detected by the naked eye. The vials were carefully analyzed, and the number of nucleated samples was counted. Then, the nucleation probability (*p*_nucleation_) was determined as the ratio of nucleated samples to the total number of samples that were irradiated.

### 2.2 Laser Pulse Position Experiments

A similar experimental approach was used to study the effect of the irradiation position on the nucleation probability of aqueous potassium chloride solutions; 100 vials of potassium chloride solutions (supersaturation of *S* = 1.034, 24 °C) were prepared as previously described. The position at which the laser pulse would reach the sample vials was adjusted by changing the height of the sample holder to four different positions with respect to the distance from the air/solution interface, as illustrated in Figure 1B–E: (1) slightly above the glass/solution interface at the bottom of the vial (the distance from the air/solution interface was 0.9H, where H is the height of the liquid column); (2) at the middle of the vial with respect to the air/solution interface (0.5H); (3) slightly below the air/solution interface at the top of the vial (0.1H), and (4) directly through the meniscus of the solution. All samples were irradiated with a single pulse of the fundamental beam with a maximum peak intensity of 10 MW/cm^2^ following the sample handling procedure described above.

## 3 Results and Discussion

### 3.1 Experimental Repeatability and Statistical Analysis

In order to check the repeatability of the NPLIN experiments, the entire experimental procedure, from sample preparation to sample checking, was performed in triplicate. Samples containing an aqueous potassium chloride solution with a supersaturation of *S* = 1.034 were irradiated in the middle of the vial with a maximum peak intensity of 10 MW/cm^2^ and a beam diameter of 9 mm.

**Figure 2:**
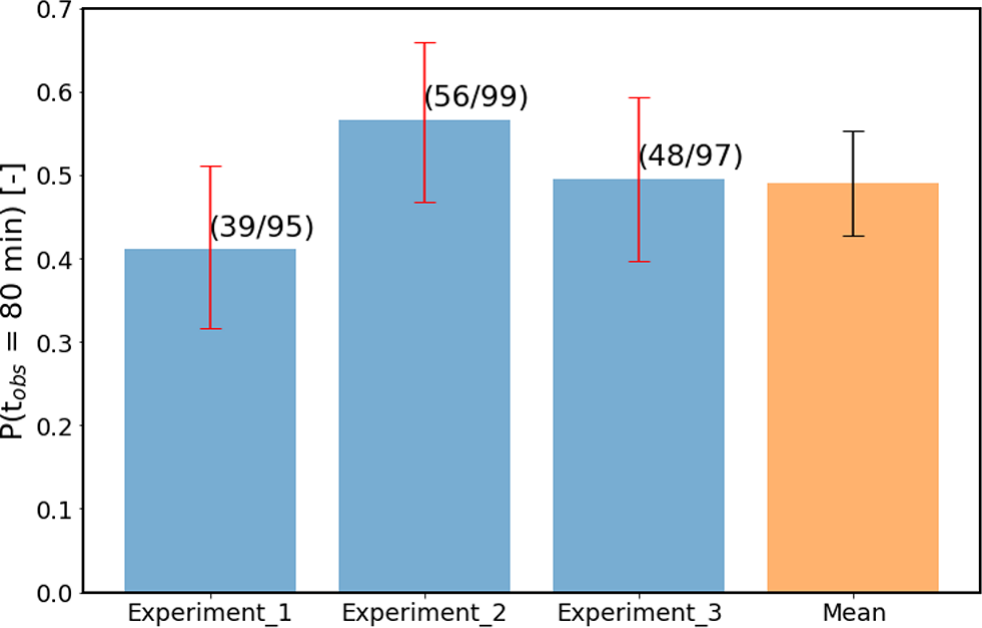
Nucleation probability in three consecutive NPLIN experiments under identical laser parameters at a fixed supersaturation of 1.034. The arithmetic mean of the three experiments is shown in orange. The number of nucleated samples and total number of samples are given next to error bars in parentheses.

Figure 2 shows a bar plot containing the results in terms of the nucleation probability per experiment. In total 39, 56, and 48 samples were nucleated out of a set of 95, 99, and 97 samples, respectively. This led to the respective nucleation probabilities of 0.41, 0.57, and 0.49. The arithmetic mean of the three nucleation probabilities was calculated to be 0.49 with a standard deviation of 0.06, as shown in Figure 2 with a black error bar.

In light of the laborious nature of the current experimental procedure, performing every experiment in triplicate is very time-consuming. Hence, throughout the rest of this study, the experimental error in NPLIN experiments was approximated by calculating statistical (95%) confidence intervals using the Wilson score method. By applying the Wilson score method to the data obtained for the repeated experiments, it was found that the widths of the confidence intervals were 0.19, 0.19, and 0.20 for experiments 1, 2, and 3, respectively (shown in Figure 2 as red error bars). Thus, the statistical error calculated from a single set of observations was found to be significantly larger than the experimental error computed from the outcome of repeated experiments (0.12, 2× the standard deviation). Judging solely from the size of the errors computed by the Wilson score method, the nucleation probabilities observed in the repeated experiments in Figure 2 are statistically identical.

### 3.2 Laser-Exposed Volume Dependency of the Nucleation Probability

**Figure 3:**
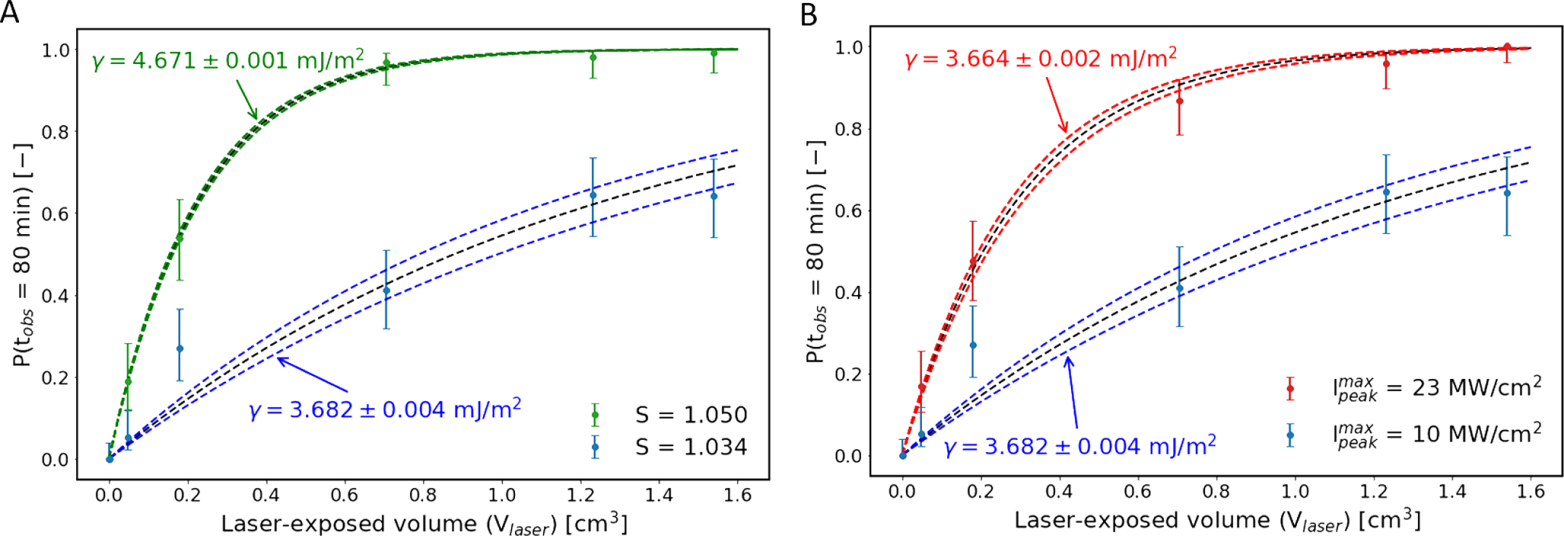
Effect of laser-exposed volume on the nucleation probability at (A) a constant maximum peak intensity of 10 MW/cm^2^ for two distinct supersaturation values and at (B) a constant supersaturation of 1.034 for two distinct peak intensities. Error bars were computed using the Wilson score method. Fits have been constructed following the DP model.

The effect of the laser-exposed volume of the solution was studied by varying the laser beam diameter using a homemade Galilean telescope, as illustrated in Figure 1. Theoretical beam diameters were obtained using the lens configurations given in Table 1. Using these values, the laser-exposed volume of the solution for each experiment was derived, assuming a refractive index of the sample^33^ (see the Supporting Information, Section 2).^34^ The effects of changing the supersaturation at a constant maximum peak intensity of 10 MW/cm^2^ are shown in Figure 3A. The effects of changing the maximum peak intensity at a constant supersaturation of 1.034 are shown in Figure 3B.

Several observations can be made from the data shown in Figure 3. First, by increasing the laser-exposed volume of the solution, an increase in the nucleation probability is observed. To the best of our knowledge, this is the first report in the literature identifying the exposed volume as an experimental parameter influencing the NPLIN probability. Second, the extent to which the nucleation probability increases as a result of the increasing laser-exposed volume is influenced by both the degree of supersaturation of the solution and the magnitude of the maximum peak intensity of the laser.

To better understand these experimental observations, a mathematical basis was constructed by using a modified DP model. Under the constraints of constant intensity and supersaturation, the average number of crystals produced, *N*_crystal_, is predicted to be proportional to the volume of the laser beam, *V*_laser_: *N*_crystal_ = *m*(*I*, *S*)*V*_laser_, where *m*(*I*, *S*) serves as an intensity and supersaturation-dependent lability constant given by
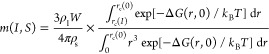
1where ρ_l_ is the density of the surrounding medium, *W* is the solute mass fraction, ρ_s_ is the density of the solute molecule, *k*_B_ is the Boltzmann constant, *T* is the temperature of the solution, *S* is the supersaturation ratio of the solution, *ΔG*(*r*, *I*) is the free energy barrier to form a cluster of radius *r* at laser intensity *I*, *r*_c_(0) is the critical cluster radius at a laser intensity of 0, and *r*_c_(*I*) is the critical cluster radius at a laser intensity *I*. The critical cluster radius *r*_c_(*I*) and free energy barrier *ΔG*(*r*, *I*) under the influence of an electric field is given by
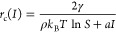
2
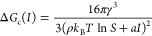
3where γ is the interfacial tension between the cluster and surrounding solution and the constant *a* contains the dielectric contrast between the solute cluster and the surrounding medium.^29^*a* is given by
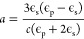
4The NPLIN nucleation probability can then be computed as a function of the lability constant (*m*) and laser-exposed volume (*V*_laser_) assuming a Poisson distribution, which is given by eq 5.^23^ This model can be used to fit experimental data without having to correct for an experimental intensity threshold. The curves shown in Figure 3 were constructed by fitting the parameter *m*(*I*, *S*) in eq 5 using a nonlinear least squares regression. The only parameter that is not estimated in this analysis is the interfacial tension between the cluster surface and the surrounding medium, γ, which is present as a function of *r*_c_ in the limits of the integrals in eq 1. Hence, by numerically solving eq 1, a phenomenological value for the interfacial tension can be derived. An overview of the physical parameters used for the calculations is given in Table 2. The values for the dielectric constant of a cluster of solute molecules, ϵ_p_, and that of the surrounding medium, ϵ_s_, have been computed by squaring the refractive indices of solid potassium chloride (*n*_KCl_ = 1.4940)^35^ and the sample solution, respectively.^36^ The complete derivation of the laser-exposed volume dependency of the nucleation probability using the modified DP model is shown in the Supporting Information, Section 1.^34^
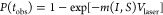
5

**Table 2:** Physical Parameters Used to Derive the Phenomenological Value of the Interfacial Tension^a^

supersaturation *S*	solute mass fraction *W*	solid density ρ_s_ (kg/m^3^)	solution density ρ_l_ (kg/m^3^)	solution refractive index *n*_3_	rel. permittivity solid ϵ_p_	rel. permittivity solution ϵ_s_	molar mass *M* (g/mol)
1.034	0.2698	1984	1184	1.3758	2.232	1.893	74.55
1.050	0.2729	1984	1184	1.3763	2.232	1.894	74.55

a Parameters are assumed to be properly estimated at a temperature of 24 °C.

Table 3 provides an overview of the parameters derived from the DP model and the numerical values computed by applying the best-fit value for the interfacial tension to the classical nucleation theory. By observing both plots in Figure 3, it was found that, in the case of increasing supersaturation or higher maximum peak intensity, the samples become more labile to nucleation and hence show a higher lability constant.

**Table 3:** Derived Parameters Using the Dielectric Polarization Model and the Parameters Computed by Applying the Value for the Interfacial Tension to the Classical Nucleation Theory

supersaturation *S*	max. peak intensity *I*_peak_^max^ (MW/cm^2^)	lability constant *m*(*I*, *S*) (cm^–3^)	interfacial tension γ (mJ/m^2^)	nucleation barrier height *ΔG*_c_(0)/*k*_b_*T*	critical radius *r*_c_(0) (nm)	dielectric free energy *ΔG*_EF_/*k*_B_*T*	Difference in critical radii *r*_c_(0) – *r*_c_(*I*) (nm)
1.034	10	0.787 (±0.088)	3.674 _(−0.003)_^(+0.004)^	41.9	3.34	–4.07 × 10^–3^	1.62 × 10^–4^
1.034	23	3.352 (±0.202)	3.656 _(−0.002)_^(+0.002)^	41.2	3.33	–9.22 × 10^–3^	3.71 × 10^–4^
1.050	10	4.367 (±0.114)	4.660 _(−0.001)_^(+0.001)^	40.2	2.91	–2.66 × 10^–3^	0.96 × 10^–4^

From the fitted lability constants, phenomenological values for the interfacial tension were computed. Asymmetrical errors were calculated due to the nonlinear relation between the lability constant and the interfacial tension. The absolute values of the interfacial tension observed are of the same order of magnitude as the values derived from previous intensity dependent NPLIN experiments that were conducted on similar aqueous potassium chloride systems by Alexander et al.^23^ (γ = 2.19 mJ/m^2^), Ward et al.^37^ (γ = 5.283 mJ/m^2^), and Fang et al.^38^ (γ = 3.16 mJ/m^2^).

Although the DP model can be employed to yield quantitative information from the current NPLIN experiments, using the fitted value of the interfacial tension to compute the absolute values of the nucleation barrier height and critical radius from the classical nucleation theory reveals ambiguous results. For all of the conducted experiments, a classical nucleation barrier of *ΔG*_c_(0) = 40*k*_B_*T* – 42*k*_B_*T* with a critical radius of *r*_c_ = 2.91 – 3.34 nm was calculated. According to these calculations and in combination with the electric field strength of the laser, the classical nucleation barrier is only lowered by a minuscule amount (from *ΔG*_EF_ = −9.22 × 10^–3^*k*_B_*T* to −2.66 × 10^–3^*k*_B_*T*). Hence, the resulting decrease in the critical radius is on the order of 1 × 10^–4^ nm for all three experiments. These results indicate that, while the DP model in other NPLIN experiments provided useful information,^8,10^ the effect of the laser electric field on the nucleation process is relatively small.

If the nucleation rate, *J*, is expressed in the form of an Arrhenius equation^39^
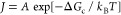
6where *A* is the pre-exponential factor (which is assumed to be constant for spontaneous and laser-induced nucleation experiments), then the nucleation rate from laser-induced experiments, *J*_laser_, should relate to the spontaneous nucleation rate, *J*_spontaneous_, by
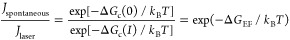
7where *ΔG*_EF_ is the change in free energy due to the introduction of an electric field (*ΔG*_EF_ = *vaI*). Here, *v* is the volume of the precritical cluster. Thus, by substituting the values (*ΔG*_EF_ = −9.22 × 10^–3^*k*_B_*T* to −2.66 × 10^–3^*k*_B_*T*) found in this study, the laser-induced nucleation rate should increase by a factor of 1.003 to 1.009 compared to the spontaneous nucleation rate.

However, according to classical nucleation theory,^39^ the nucleation rate (*J*) is inversely proportional to the induction time (*t*_ind_).^39^ Thus, laser-induced nucleation should increase at least a hundred-fold compared to spontaneous nucleation when under similar experimental conditions. Consistent with this theory, the spontaneous nucleation of aqueous potassium chloride solutions typically takes 1–2 weeks under such conditions. However, Kacker et al.^40^ have shown that nucleation can occur within 80 min in laser-induced experiments, indicating a significant increase in the nucleation rate. Unless laser irradiation of the samples plays a significant role in the value of the pre-exponential factor, the massive decrease in nucleation time cannot be explained quantitatively in the context of the DP model, thus highlighting the limitations of the current approach. Ward et al.^37^ explained similar ambiguous results by introducing the two-step nucleation model. It was argued that the low electric field strength might play a significant role in structurally reorganizing the amorphous liquid-like clusters, lowering the second nucleation barrier and hence accounting for the observations made from NPLIN experiments.^37^ However, it proved to be challenging to obtain quantitative evidence to substantiate such a claim. We acknowledge that the proposed two-step nucleation explanation may also be valid for our observations. However, providing supporting experimental evidence is beyond the scope of the current work.

Alternatively, the experimental findings could be explained qualitatively through the nanoparticle heating mechanism. By increasing the laser beam diameter, a larger volume of the solution is irradiated. If it is assumed that the nanoparticles are homogeneously suspended throughout the sample volume, this inevitably means that the number of nanoparticles irradiated increases with an increasing beam diameter. This, in turn, would lead to an increased number of vapor cavities and more locally increased supersaturations, from which an increase in the nucleation probability would be expected. The increase in the supersaturation of the solution would lead to higher locally supersaturated regions upon laser irradiation with similar conditions, resulting in a higher nucleation probability. Likewise, an increased laser intensity is expected to create larger vapor cavities due to the more extensive heating of the nanoparticles. This, again, would result in higher locally supersaturated regions and the subsequent increase of the probability of nucleation. Although the nanoparticle heating mechanism provides the basic qualitative explanation for the current experimental observations, further research that examines various nanoimpurity size distributions and impurity compositions could provide a more in-depth understanding of this mechanism through experiments.

### 3.3 Crystal Morphology and Number of Crystals per Nucleated Sample

**Figure 4:**
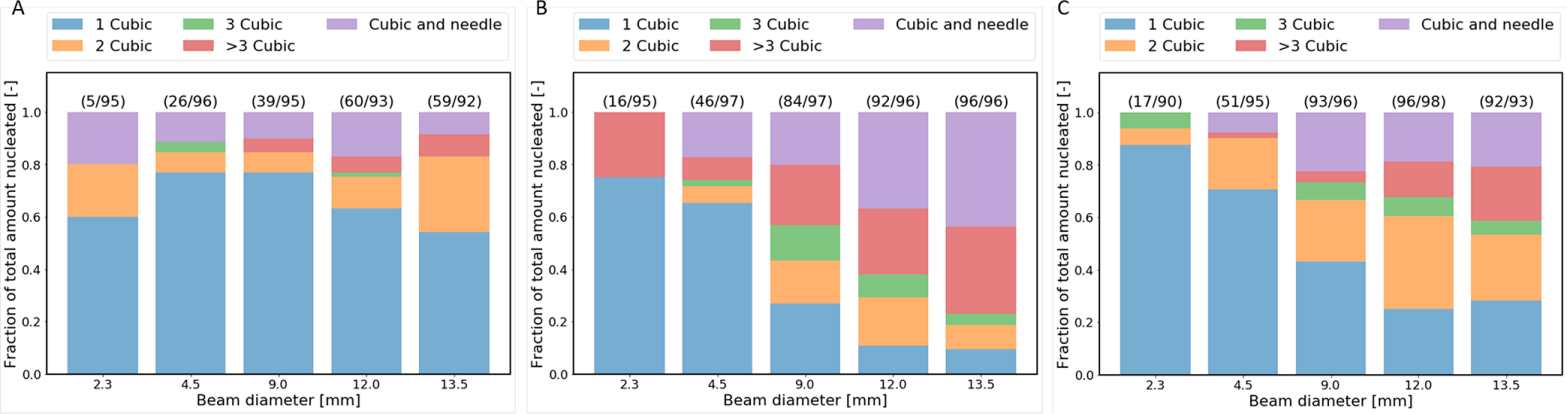
Bar plots showing the relative number of crystals per nucleated sample in experiments on aqueous potassium chloride solutions under distinct supersaturations, S, and maximum laser peak intensities, *I*_peak_^max^. (A) *S* = 1.034 and *I*_peak_^max^ = 10 MW/cm^2^, (B) *S* = 1.050 and *I*_peak_^max^ = 10 MW/cm^2^, and (C) *S* = 1.034 and *I*_peak_^max^ = 23 MW/cm^2^. The number of samples nucleated and the total number of samples irradiated per experiment are shown in parentheses above the corresponding bar.

In addition to studying the number of samples nucleated in an effort to relate the nucleation probability to the laser-exposed volume of the solution, we also report the number of crystals and type of crystal morphology per experiment in this study. Figure 4 shows the distribution of the number of crystals and crystal geometries per nucleated sample for each laser-exposed volume experiment.

In general, the following observations can be made from Figure 4. The combination of a low supersaturation (*S* = 1.034) and a low maximum peak intensity (*I*_peak_^max^ = 10 MW/cm^2^) resulted in predominantly single cubic crystals for each of the beam diameters (see Figure 4A). Some accounts of two cubic crystals per nucleated sample were observed as well as the combination of cubic and needle-like crystals. However, no clear relationship between the amount of crystals per sample and the laser beam diameter was found. Increasing the supersaturation of the aqueous potassium chloride solutions revealed a general increase in the number of crystals per irradiated/nucleated sample as the beam diameter increased (see Figure 4B). The same effect was observed by increasing the maximum peak intensity (see Figure 4C), which is in accordance with previous reported experiments on aqueous potassium chloride solutions under similar conditions.^23,37,40^ Apart from the relationship between the number of crystals per nucleated sample and increasing the maximum peak intensity of the laser, the use of large beam diameters in combination with high maximum peak intensities clearly favors the formation of a large number of cubic crystals (≥3 cubic) or mixtures of cubic and needle-shaped crystals.

Increasing the laser beam diameter and the maximum peak intensity or bulk supersaturation can lead to the formation of more nucleation sites, as a larger area of the solution is exposed to the laser beam. This can result in the formation of multiple cubes or a mixture of cubes and needles due to the increased number of nucleation sites and the decreased amount of solute available for each nucleus.^41^

### 3.4 Effect of Irradiation Position on NPLIN Probability

The experimental configuration in the experiments addressing the effect of irradiation position of the NPLIN probability is shown in Figure 3B–E. No significant change in the nucleation probability was observed when the unfocused incident laser beam passed through the bottom, top, or middle positions of the cylindrical vial. In contrast, laser irradiation through the meniscus of the solution yielded an increase in the nucleation probability by approximately 40–50%.

At the bottom, middle, and top positions, there is no change in the solution’s refractive index or in the geometry of the sample container. Thus, the laser beam is expected to act identically with the solution volume at these positions. By assuming a homogeneous distribution of precritical clusters and considering that the laser-exposed volume is the same for the bottom, middle, and top positions, no significant change in the nucleation probability is expected according to the DP model. The observations of the position-dependent experiments can also be explained similarly in the context of the nanoparticle heating mechanism. Again, by taking into account that the laser acts identically on the solution volume at the bottom, middle, and top positions and by assuming a homogeneous distribution of nanoparticles/nanoimpurities throughout the sample volume, no change in the nucleation probability is expected.

In an attempt to better understand the nucleation probability results and the laser focusing effect at the meniscus, a ray tracing simulation using the Zemax OpticStudio software (v.23.2.01) was performed, as analytical estimates proved challenging. Geometries were created with dimensions identical to those of an HPLC vial (BGB, dimensions of 61 × 16.6 mm) using the CAD functionality tool. Glass, an aqueous KCl solution, and air, with individual refractive indices of 1.5, 1.4940, and 1, respectively, were assigned as materials within the vial to carry out the simulation. To understand how light rays operate when interacting with the meniscus in our system, the concept of total internal reflection becomes important. Governed by Snell’s law, total internal reflection occurs when a light ray traversing a medium of a higher refractive index meets a boundary of a medium with a lower refractive index at an angle greater than the critical angle, resulting in the ray reflecting back. In this context, the critical angle is approximately 42°, derived from ϕ = arcsin(1/1.494), as light rays travel from the solution medium to the air medium at the meniscus. The concave nature of the meniscus and the collimated nature of the incident laser beam implies that most rays reach the air/solution boundary at an angle larger than this critical value, resulting in a downward reflection (total internal reflection) of the light rays, as can be seen in the Supporting Information, Figure SVA,B (side view and top view, respectively). As the Gaussian laser beam (9 mm in diameter) with a peak intensity of 5 MW/cm^2^ irradiates the supersaturated solution horizontally, the HPLC vial acts analogously to a cylindrical lens, focusing the beam behind it. Meanwhile, the concave meniscus of the solution and the rear wall of the vial reflect the incoming light rays into the solution medium following total internal reflection. The overlap of these two optical phenomena leads to a significant accumulation of the laser peak intensity just below the meniscus. At this stage, in order to acquire a comprehensive understanding of the laser intensity distribution beneath the meniscus in these simulations, a volume detector was placed directly below it with the bottom region of the meniscus encompassed (the orange colored rectangle) within the detecting volume. The volume detector was positioned based on the location of the experimentally observed crystal formation on the meniscus following the laser shot. The intensity distribution was then visualized using two-dimensional slices from the three-dimensional volumetric detector. Specific visual representations across the *XY* and *YZ* planes, along with the associated laser peak intensity at a particular coordinate as an example, are detailed in the Supporting Information, Figure SVC,D.

Possible scenarios that could explain the increased nucleation probability at the meniscus within the context of the DP model include (1) the preferential adsorption of the solute onto the air/solution surface, which suggests a higher number of critical clusters at the interface, and (2) the observed intricate refraction of the laser beam due to the complex vial geometry and meniscus curvature creating areas with higher laser intensities at the meniscus, thereby lowering the nucleation barrier by a greater amount and changing the outcome of the laser-induced nucleation experiments compared to the other irradiation positions. Similarly, from the perspective of the nanoparticle heating mechanism, an increase in the peak intensity at the meniscus could lead to more intense light absorption by the nanoparticles/nanoimpurities at the air/solution interface, thus altering the vapor cavity radius and enhancing supersaturation.

From the perspective of the nanoparticle heating mechanism, an additional scenario arises. At the air/solution interface, dust particles may adhere and, hence, locally increase the “impurity” concentration, which has been shown to have an increasing effect on the nucleation probability in previous NPLIN experiments.^26,27,42^

Previous reports explaining the increase of the nucleation probability at the air/solution interface have been provided in the literature. Ikni et al.^16^ and Liu et al.^43^ observed an increase in the crystallization probability at the air/solution interface with respect to carbamazepine and glycine supersaturated solutions irradiated with a femtosecond laser and a nanosecond laser, respectively. The authors attributed this observation to the interplay between molecular adsorption and surface deformation, resulting in a distinct solution flow from the surface due to its free boundary characteristic. This unique flow resulting from the surface deformation might contribute to enhancing the crystallization probability at the air/solution (meniscus) interface. Similarly, Clair et al.^44^ observed that glycine crystals obtained through NPLIN nucleate at the meniscus and exhibit different morphologies, even when the laser was directed through the air/solution interface from above.

**Figure 5:**
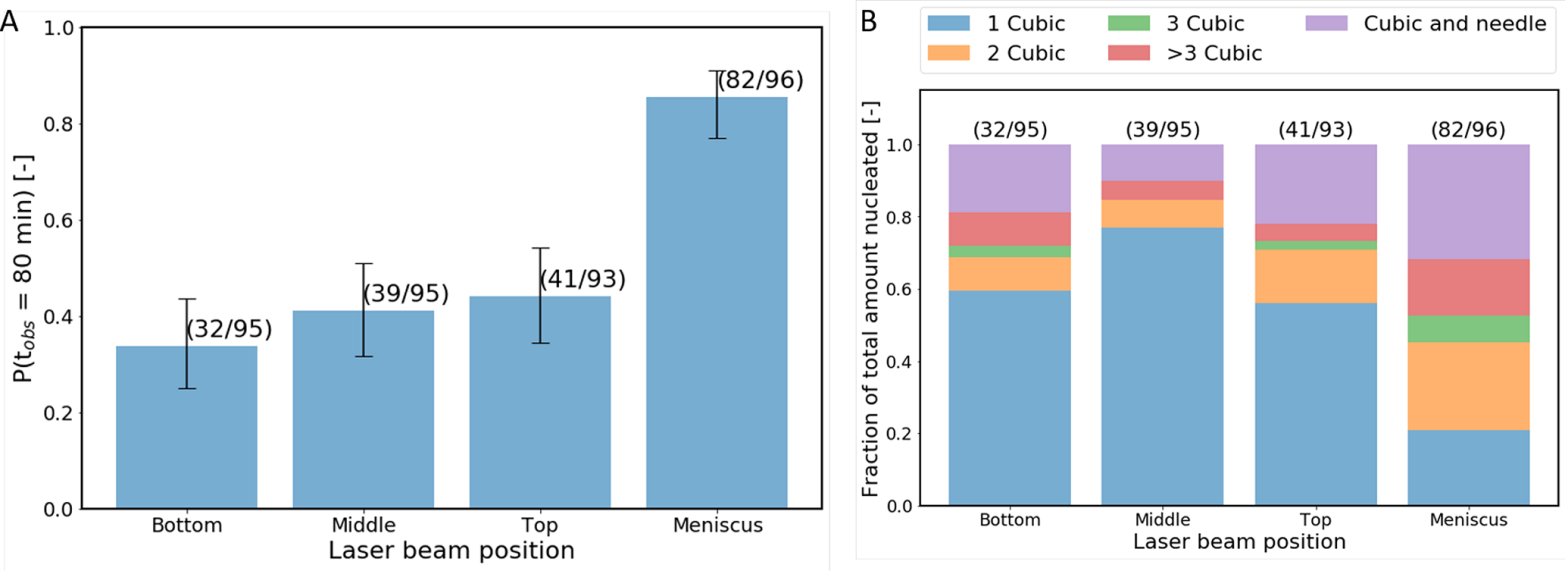
Bar plots showing the nucleation probability for different positions of the laser beam with respect to the interfaces within the glass vials. Experiments were performed on aqueous potassium chloride solutions (*S* = 1.034) using a maximum peak intensity of 10 MW/cm^2^. Error bars were computed using the Wilson score method. The number of samples nucleated and the total number of samples irradiated per experiment are shown in parentheses above the corresponding bar.

Figure 5B provides an overview of the relative amount of crystals (and different crystal morphologies) per nucleated sample in the position experiments. The bottom, middle, and top experiments show a dominance in single crystals per nucleated sample, and no clear distinction in the crystal number or morphology could be observed. In contrast, in the experiments in which the meniscus of the solution was irradiated, a significant increase in the number of crystals was observed per nucleated sample, as well as an increase in the combination of cubic and needle-like geometries. These results support the presence of multiple nucleation sites at the meniscus, caused either by an increase in the generated supersaturation due to irradiation,^45^ by multiple peak intensity hot spots, or from dust adhesion to the surface, thus leading to heterogeneous nucleation. A similar increase in the number of crystals for KCl supersaturated solutions with an increase in the laser peak intensity was also observed in the literature.^46,47^ Hua et al.^47^ studied microfluidic laser-induced nucleation in supersaturated KCl solutions with supersaturations ranging from 1.06 to 1.10 and observed that the number of crystals that formed in the microfluidic device was proportional to the laser intensity. Meanwhile, Duffus et al.^46^ observed crystal nucleation behavior in KCl–agarose gels prepared using 0.12–0.75% w/w powdered agarose in 1.06 supersaturated KCl solutions. Their findings also indicated a direct relationship between laser peak intensity and crystal formation.

## 4 Conclusions

This study focused on how the NPLIN probability of supersaturated aqueous potassium chloride solutions is influenced by the laser-exposed volume and laser position. Despite their significance in the industrial-scale implementation of NPLIN, these topics have not been extensively discussed in the NPLIN literature, if at all.

The NPLIN probability was found to depend on the laser-exposed volume under the constraints of constant supersaturation and peak intensity. An increase in the number of crystals per nucleated sample was also observed by enlarging the laser-exposed volume. Both the nanoparticle heating and dielectric polarization models were able to partly explain these observations. However, in the absence of data that exclusively favor one of these models, we cannot definitively conclude which mechanism is dominant in the presented experiments. Further experiments exploring the effects of different nanoparticles (size and concentration) on the nucleation probability could provide valuable insights into the underlying mechanisms of NPLIN.

Regarding the effect of laser position, irradiation not near the interfaces or in the vicinity of the air/solution or glass/solution interfaces yielded no change in the nucleation probability. A significant increase in the nucleation probability was observed only for irradiation directly through the air/solution interface (meniscus). This observation was accompanied by an increase in the number of crystals observed per sample, along with an increase in cubic and needle-like geometries. This is attributed to preferential adsorption at the interface, an increased laser peak intensity at the meniscus caused by complex laser refraction, or the laser-induced heating of dust/impurity particles adhered at the interface, resulting in evaporation. The presented results will contribute to the rational design of potential industrial applications of NPLIN, where controlling crystal quality parameters, such as morphology, is of paramount importance.^48,49^

## Supporting Information Available

Details of the laser-exposed volume dependency of the DP model, the laser-exposed volume calculation, and laser operation and choice of laser parameters, and the Zemax OpticStudio simulation results (PDF)
