# Supplementary material for: Effect of Laser-Exposed Volume and Irradiation Position on Nonphotochemical Laser-Induced Nucleation of Potassium Chloride Solutions
Source: Cryst Growth Des. 2023 Oct 16;23(11):8163–72. doi: 10.1021/acs.cgd.3c00865 (PMC10626568; doi:10.1021/acs.cgd.3c00865)
Supplement: Supplementary file 1 — cg3c00865_si_001.pdf [file cg3c00865_si_001.pdf]

Supplementary Information: Effect of Laser Exposed Volume and Irradiation Positionon Non-Photochemical Laser-Induced Nucleation of Potassium Chloride Solutions

Vikram Korede,† Mias Veldhuis,† Frederico Marques Penha,‡ Nagaraj Nagalingam,† PingPing Cui,¶ Antoine E.D.M. van der Heijden,† Herman J.M. Kramer,† and Hüseyin Burak Eral*,†

†*Process & Energy Department, Delft University of Technology, Leeghwaterstraat 39, 2628 CB Delft, The Netherlands*

‡*Department of Chemical Engineering, KTH Royal Institute of Technology, Teknikringen 42, 114-28 Stockholm, Sweden*

¶*School of Chemical Engineering and Technology, State Key Laboratory of Chemical Engineering, Tianjin University, Tianjin 300072, People's Republic of China.*

E-mail: h.b.eral@tudelft.nl

1 Laser Exposed Volume Dependency of DP Model

In line with the Dielectric Polarization model, the free energy change of cluster formation, ΔG, is made up of contributions from the free energy change of surface formation, ΔG_s, the free energy change of phase transformation, ΔG_v, and the change in free energy due to the introduction of an electric field, ΔG_{EF}. In presence of an electric field and under the constraint that the $\epsilon_p > \epsilon_s$, the free energy change of cluster formation (ΔG) is lowered by an amount proportional to $-v(\epsilon_p - \epsilon_s)E^2$, where v is the volume of the cluster, ϵ_p is the dielectric constant of a cluster of solute molecules, ϵ_s is the dielectric constant of the surrounding medium and E is the electric field strength.[1] For convenience, the electric field strength can be written in terms of intensity by the equation $I = \frac{1}{2}\epsilon_0 c E^2$, where I is the intensity of the light, ϵ_0 is the vacuum permittivity and c is the speed of light. Hence, by adding the contribution of the electric field induced by the light, the change in free energy becomes,

$$\begin{aligned}\Delta G &= \Delta G_s + \Delta G_v + \Delta G_{EF} \\ &= s\gamma + v\rho\Delta\mu - vaI\end{aligned} \tag{1}$$

where s and v are the surface area and volume of the precritical cluster, respectively, γ is the interfacial tension between the cluster and surrounding solution, ρ is the number of molecules per unit volume in the solid phase and $\Delta\mu$ is difference in chemical potential of the substance in solution and in the crystal. The constant a contains the dielectric contrast between solute cluster and the surrounding medium,[?]

$$a = \frac{3\epsilon_s(\epsilon_p - \epsilon_s)}{c(\epsilon_p + 2\epsilon_s)} \tag{2}$$

Then, assuming the precritical clusters are spheres with a radius r, the free energy change of cluster formation becomes,

$$\Delta G(r, I) = 4\pi r^2\gamma + \frac{4}{3}\pi r^3\Delta\mu - \frac{4}{3}\pi r^3 aI \tag{3}$$

Substituting chemical potential in terms of supersaturation in equation 3, assuming a supersaturated solution in which the solute chemical potential exceeds the chemical potential of the crystal, results in,

$$\Delta G(r,I) = 4\pi r^2\gamma - \frac{4}{3}\pi r^3(\rho k_B T \ln S + aI) \quad (4)$$

where k_B is the Boltzmann constant, T is the temperature of the solution and S is the supersaturation ratio of the solution. Analogously to the Classical Nucleation Theory it is possible to derive the critical radius, $r_c(I)$, and nucleation barrier height, $\Delta G_c(I)$, under the influence of an electric field,

$$r_c(I) = \frac{2\gamma}{\rho k_B T \ln S + aI} \quad (5)$$

$$\Delta G_c(I) = \frac{16\pi\gamma^3}{3(\rho k_B T \ln S + aI)^2} \quad (6)$$

Prior to laser irradiation when nucleation has not yet occurred, all clusters are said to be smaller than the critical radius ($r < r_c(0)$). Upon laser irradiation, all clusters with size $r_c(I) < r < r_c(0)$ become supercritical. The average number of precritical clusters ($r < r_c(0)$) can be computed by,

$$N_{cluster} = \frac{N_{molecule}}{\langle n \rangle} \quad (7)$$

where $N_{molecule}$ is the number of solute molecules in the volume of the laser beam, and $\langle n \rangle$ is the average amount of solute molecules in a precritical cluster, which can be computed by assuming a Boltzmann distribution over the domain $r \in [0, r_c(0)]$,

$$\begin{aligned}\langle n \rangle &= \frac{4\pi\rho \langle r^3 \rangle}{3} \\ &= \frac{4\pi\rho}{3} \times \frac{\int_0^{r_c(0)} r^3 \exp\left[-\Delta G(r,0)/k_B T\right] dr}{\int_0^{r_c(0)} \exp\left[-\Delta G(r,0)/k_B T\right] dr}\end{aligned} \quad (8)$$

Hence,

$$N_{cluster} = \frac{3N_{molecule}}{4\pi\rho} \times \frac{\int_0^{r_c(0)} \exp\left[-\Delta G(r,0)/k_B T\right] dr}{\int_0^{r_c(0)} r^3 \exp[-\Delta G(r,0)/k_B T] dr} \quad (9)$$

To obtain the average number of clusters that go on to form viable crystals after laser irradiation, $N_{crystal}$, the average amount of precritical clusters is multiplied by the fraction of clusters that become supercritical,

$$N_{crystal} = N_{cluster} \times \frac{\int_{r_c(I)}^{r_c(0)} \exp\left[-\Delta G(r,0)/k_B T\right] dr}{\int_0^{r_c(0)} \exp[-\Delta G(r,0)/k_B T] dr} \quad (10)$$

Hence, by combining equation 9 and 10 a function for the average amount of clusters that go on to form viable crystals is obtained,

$$N_{crystal} = \frac{3N_{molecule}}{4\pi\rho} \times \frac{\int_{r_c(I)}^{r_c(0)} \exp\left[-\Delta G(r,0)/k_B T\right] dr}{\int_0^{r_c(0)} r^3 \exp[-\Delta G(r,0)/k_B T] dr} \quad (11)$$

The amount of solute molecules per unit volume can be written in terms of the solute density, $\rho = \frac{\rho_s N_A}{M}$, where N_A is Avogadro's constant and M is the molar mass of the solute. In addition, the amount of molecules in the volume of the laser beam can be written as,

$$N_{molecule} = \frac{V_{laser}\rho_l N_A W}{M} \quad (12)$$

where V_{laser} is the volume of the laser passing through the solution,ρ_l is the density of the surrounding medium and W is the solute mass fraction. Therefore the average amount of clusters that go on to form viable crystals becomes,

$$N_{crystal} = \frac{3V_{laser}\rho_l W}{4\pi\rho_s} \times \frac{\int_{r_c(I)}^{r_c(0)} \exp\left[-\Delta G(r,0)/k_B T\right] dr}{\int_0^{r_c(0)} r^3 \exp[-\Delta G(r,0)/k_B T] dr} \quad (13)$$

Under the constraints of constant intensity and

supersaturation, the only dependent variable is V_{laser}, and hence we can write $N_{crystal}$ as,

$$N_{crystal} = m(I,S)V_{laser} \tag{14}$$

where $m(I,S)$ serves as a new, intensity and supersaturation dependent lability constant,

$$m(I,S) = \frac{3\rho_l W}{4\pi\rho_s} \times \frac{\int_{r_c(I)}^{r_c(0)} \exp\left[-\Delta G(r,0)/k_B T\right]dr}{\int_0^{r_c(0)} r^3 \exp[-\Delta G(r,0)/k_B T]dr} \tag{15}$$

By assuming a Poisson distribution it is possible to compute the probability that no crystals are observed in any of the repeated experiments,

$$p_0 = \exp[-m(I,S)V_{laser}] \tag{16}$$

At last, the cumulative nucleation probability is computed as a function of intensity, supersaturation and laser exposed volume,

$$\begin{aligned} p_{nucleation} &= 1 - p_0 \\ &= 1 - \exp[-m(I,S)V_{laser}] \end{aligned} \tag{17}$$

2 Laser Exposed Volume Calculation

A cross-sectional view in the xy-plane of the laser beam passing through the vial is shown in Figure SIA. The laser passes through three media with different refractive indices, resulting in two directional changes of the laser path in the y-direction. In order to calculate the volume of the beam, the angles of incidence and angles of refraction passing through the vial have to be calculated. The angle of incidence resulting from the change of medium from air to borosilicate glass, Θ_1, can be computed by,

$$\sin\Theta_1 = \frac{r}{R_0} \tag{18}$$

where, r is the radius of the laser beam at a given cross-section and R_0 is the radius of the outer surface of the glass vial. Note that in Figure SIA, r is given as the radius of the incident beam, R_L. The usefulness of this will become evident at a later stage of this derivation. Then, by implementing Snell's law, it is possible to compute the first angle of refraction, Θ_2,

$$n_1 \sin\Theta_1 = n_2 \sin\Theta_2 \tag{19}$$

where n_1 and n_2 are the refractive indices of the air and borosilicate glass, respectively. Further inspection of a zoomed in version of the triangle enclosed by the path of the laser through the glass wall, L_L, and the outer and inner radius of the glass vial, R_0 and R_1, respectively (see Figure SIB) reveals,

$$L_L \sin\Theta_2 = R_1 \sin\Delta\Theta \tag{20}$$

where $\Delta\Theta$ is the angle between R_0 and R_1. In addition, it shows that,

$$R_0 = L_L \cos\Theta_2 + R_1 \cos\Delta\Theta \tag{21}$$

Hence, by combining equations 20 and 21 an equation is obtained to describe the outer radius of the vial as a function of $\Delta\Theta$,

$$R_0 = \frac{R_1 \sin\Delta\Theta}{\tan\Theta_2} + R_1 \cos\Delta\Theta \tag{22}$$

Equation 22 can be solved numerically to find the value for $\Delta\Theta$. Following a geometrical derivation, the second angle of incidence, Θ_3, can be written as $\Theta_2 + \Delta\Theta$. Then, using Snell's law again, the second angle of refraction, Θ_4 is obtained,

$$n_2 \sin(\Theta_2 + \Delta\Theta) = n_3 \sin\Theta_4 \tag{23}$$

From equations 18-23 all necessary angles can be computed in order to calculate the central angle, Θ_s, and subsequently the area of the disc segment, A_{chord}. The central angle is given by,

$$\Theta_s = \pi - 2\Theta_4 \tag{24}$$

and the area of the disc segment by,

$$A_{chord} = \frac{R_1^2}{2}(\Theta_s - \sin\Theta_s) \tag{25}$$

The cross-sectional area of the inner circle of

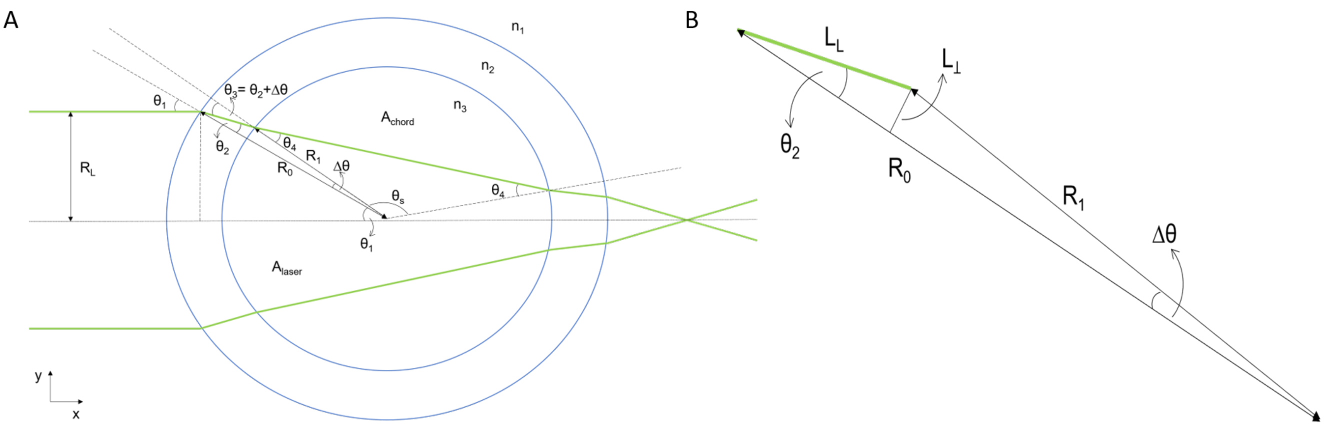

Figure SI: (A) Cross-sectional area of the laser beam passing through a cylindrical vial. This figure is not to scale and thus the given angles are only a guide to the eye, (B) Zoomed in version of the triangle enclosed by the path of the beam and the outer and inner radius of the glass vial.

the vial, A_{circle}, can be written in terms of πR_1^2. Hence, the area of the laser path through the solution, A_{laser}, becomes,

$$A_{laser} = A_{circle} - 2A_{chord} = R_1^2 \left(\pi - \Theta_s + \sin\Theta_s\right) \tag{26}$$

Subsituting equation 24 into equation 26 will give the area of the laser passing through the solution in terms of the second angle of refraction,

$$A_{laser} = R_1^2 \left(2\Theta_4 + \sin 2\Theta_4\right) \tag{27}$$

Now that the cross-sectional area of a particular slice of the laser beam has been obtained, it is possible to compute the volume of the laser beam through the solution by summing all slices of the cross-sectional area. One particular slice is defined by half a chord length of the cross-sectional area of the incident laser beam (see Figure SII). Hence,

$$r = R_L \cos\phi \tag{28}$$

Substituting equation 28 in equation 18 results in,

$$\sin\Theta_1 = \frac{R_L \cos\phi}{R_0} \tag{29}$$

Following this the area of the laser passing through the solution, A_{laser}, becomes a function of ϕ. The laser exposed volume, V_{laser}, can be computed by summing over all the slices,

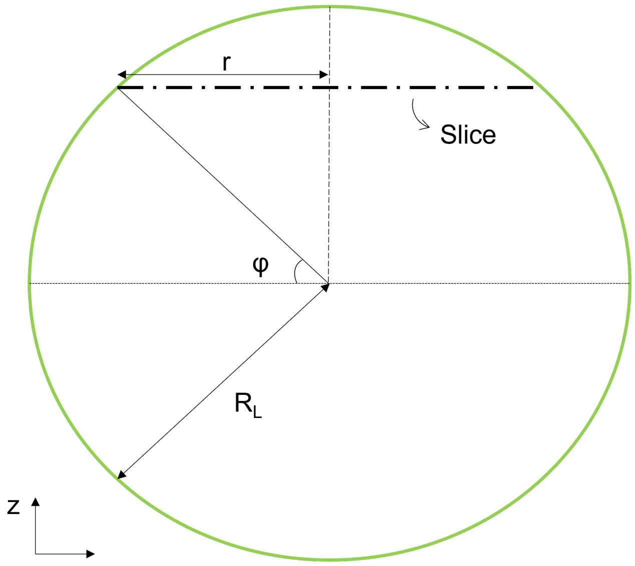

Figure SII: Cross-sectional area of the incident laser beam.

$$V_{laser} = 2 \cdot \int_0^{\frac{1}{2}\pi} A_{laser} dr \quad (30)$$

or, by substituting equation 28 and the derivative of equation 29,

$$V_{laser} = 2R_L R_1^2 \cdot \int_0^{\frac{1}{2}\pi} (2\Theta_4 + \sin(2\Theta_4)) \cos\phi d\phi \quad (31)$$

The integral in equation 31 has been solved numerically in order to find the volume of the laser beam passing through the solution. In addition, by recognizing that the cross-sectional area of the laser beam gets smaller due to the focusing effect in the y-direction, it is possible to calculate the change in intensity of the beam throughout the vial. Focusing the beam in solely the y-direction changes the profile of the beam from a circle to an ellipse. The radius of the ellipse in the y-direction is defined by the linear function of the laser beam through the solution, $y = mx + b$ (see Figure SIII). The slope coefficient, m, can be computed by,

$$m = \tan(-\Theta_m) = \tan(\Theta_1 + \Delta\Theta - \Theta_4) \quad (32)$$

At last, it is possible to compute the width of the ellipse at the end of the vial, $R_{ellipse}$,

$$R_{ellipse} = R_1 \sin(\Theta_4 - \Theta_m) \quad (33)$$

This allows for the calculation of the intensity of the laser beam at the exit of the vial.

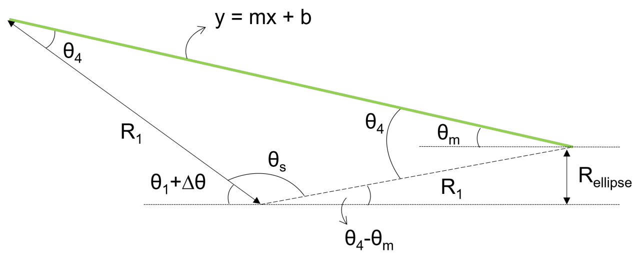

Figure SIII: Zoomed in version of the triangle enclosed by the chord formed by the path of the beam through the solution and the inner radii of the glass vial.

3 Laser operation and choice of laser parameters

Previous experiments on aqueous potassium chloride solutions have shown that the cumulative nucleation probability is a function of the peak intensity of the laser.[2?] In the current study, the peak intensity, I_{peak} is calculated by dividing the peak power, P_{peak}, by the cross-sectional area of the beam, A_{beam}.

$$I_{peak} = \frac{P_{peak}}{A_{beam}} \quad (34)$$

The peak power of the beam is obtained from the laser characteristics,

$$P_{peak} = \frac{E_{pulse}}{\tau} \quad (35)$$

where E_{pulse} is the pulse energy of the laser and τ is the pulse width. Substituting equation 35 in equation 34 and assuming that the cross-sectional area of the fundamental beam is a circle, results in

$$I_{peak} = \frac{2E_{pulse}}{\tau\pi R_L^2} \quad (36)$$

where R_L is the laser beam radius. The borosilicate HPLC vials act as a cylindrical lens due to their geometry and difference in refractive indices of air (n_1), borosilicate glass (n_2) and potassium chloride solution (n_3). As a result, the peak intensity of the laser beam changes depending on the position within the vial. Because the vials only focus the beam in the y-direction, the resulting cross-sectional area of the beam within the vial can be described by that of an ellipse, and hence the peak intensity becomes

$$I_{peak} = \frac{2E_{pulse}}{\tau\pi R_L R_{ellipse}(x)} \quad (37)$$

where $R_{ellipse}(x)$ is the length of the semi-minor axis of the ellipse, depending on the position, x, within the vial. Figure SIVA shows a cross-sectional top view in the xy-plane of the vial and corresponding fundamental laser beam

with a diameter of 9 mm. The area of the cross-section of the laser beam is smallest at the back of the vial, and hence a maximum peak intensity, I_{peak}^{max}, is found at the position where the laser hits the inner walls at the back of the vial for the first time.

The first angle of incidence (Θ_1) changes depending on the laser beam diameter. As a result, the relative change of peak intensity throughout the vial is different for each beam diameter (shown in Figure SIVB). The peak intensity becomes increasingly larger when the laser propagates throughout the vial. This effect increases the larger the incident beam diameter. To account for this difference, *a priori* calculations have been performed to determine the necessary peak intensity of the laser beam in front of the vial to achieve a maximum peak intensity that is constant for all beam diameters. Values of 16.6 and 0.5 mm were assumed for the vial outer diameter and glass thickness, respectively. In addition, the refractive index of air and borosilicate glass are assumed to be 1.0000 and 1.5195, respectively.[3] In absence of equipment to accurately record the refractive index of the potassium chloride solutions, an assumption has been made by calculating the refractive index using an empirical relation[4]

$$n_3 = 1.3352 + (1.6167{\cdot}10^{-3})c - (4.0{\cdot}10^{-7})c^2 - (1.1356{\cdot}10^{-4})T - (5.7{\cdot}10^{-9})T^2 \quad (38)$$

where c is the concentration of the solution in $g_{KCl}/g_{solution}{\times}100\%$ and T the temperature of the solution in $^{\circ}C$.

4 Zemax simulation

The additional data presented here elaborates on the laser-meniscus interaction study conducted within an HPLC vial. This is further illustrated with extended visualizations from the CAD model, which specifically emphasize the laser's interaction with the meniscus. This interaction can be seen from both the side and top views as shown in Figures SVA and SVB. In experiments, it has been observed that crystals always form at a point At this stage, to acquire a comprehensive understanding of the laser intensity distribution beneath the meniscus, a volumetric detector of dimensions 8*4*8 mm, color coded orange was placed directly below it. The volume detector was positioned based on the location of crystal formation observed experimentally on the meniscus following the laser shot. The positioning ensured that the bottom region of the meniscus was encompassed within the detecting volume. This detector was composed of 4 million voxels arranged in a 200 x 100 x 200 configuration, with each voxel designed to detect transmitted rays (flux) in MW/cm^2. Each voxel has a dimension of $40{\times}40{\times}40\mu$m. The power distribution within this 3D volume was visualized using 2D slices. For clarity and illustrative purposes, the intensity distribution is represented across both parallel (XY) and perpendicular (YZ) planes, as depicted in Figures SVC & SVD. These images show the distribution at a fixed position of Z = 0 mm and a depth of X = 0 mm (axis numbers goes from -4 mm to 4 mm), respectively. For instance, at specific coordinates (highlighted as X in the figure), the detected laser peak intensity was approximately 17.18 MW/cm^2. Such pronounced peak intensities, especially at the meniscus, could be attributed to the cylindrical nature of the vial, the total internal reflection induced by the meniscus, and the rear wall partial reflection. These factors may probably explain the high nucleation probabilities observed in laser irradiation experiments at the meniscus in the context of Dielectric Polarization model or Nanoparticle Heating mechanism.

References

(1) Alexander, A. J.; Camp, P. J. Non-photochemical laser-induced nucleation. *The Journal of chemical physics* **2019**, *150*, 040901.

(2) Ward, M. R.; Alexander, A. J. Nonphotochemical laser-induced nucleation of potassium halides: Effects of wavelength and

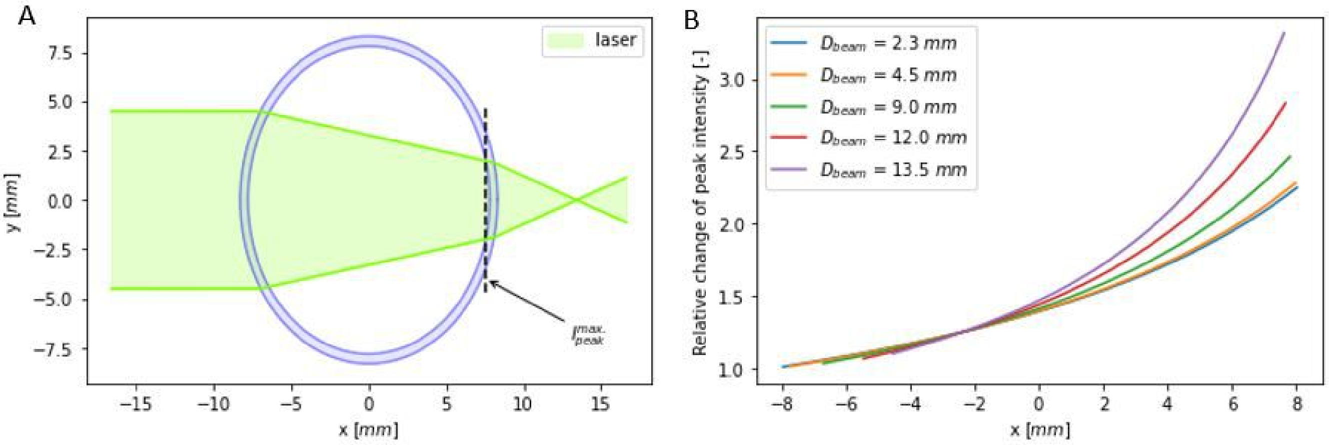

Figure SIV: (a) Top view of the laser path through a vial containing an aqueous potassium chloride solution (S = 1.034). A incident beam diameter of 9.0 *mm* is shown. (b) Plot showing relative change in peak intensity throughout the vial for different beam diameters (D_{beam}).

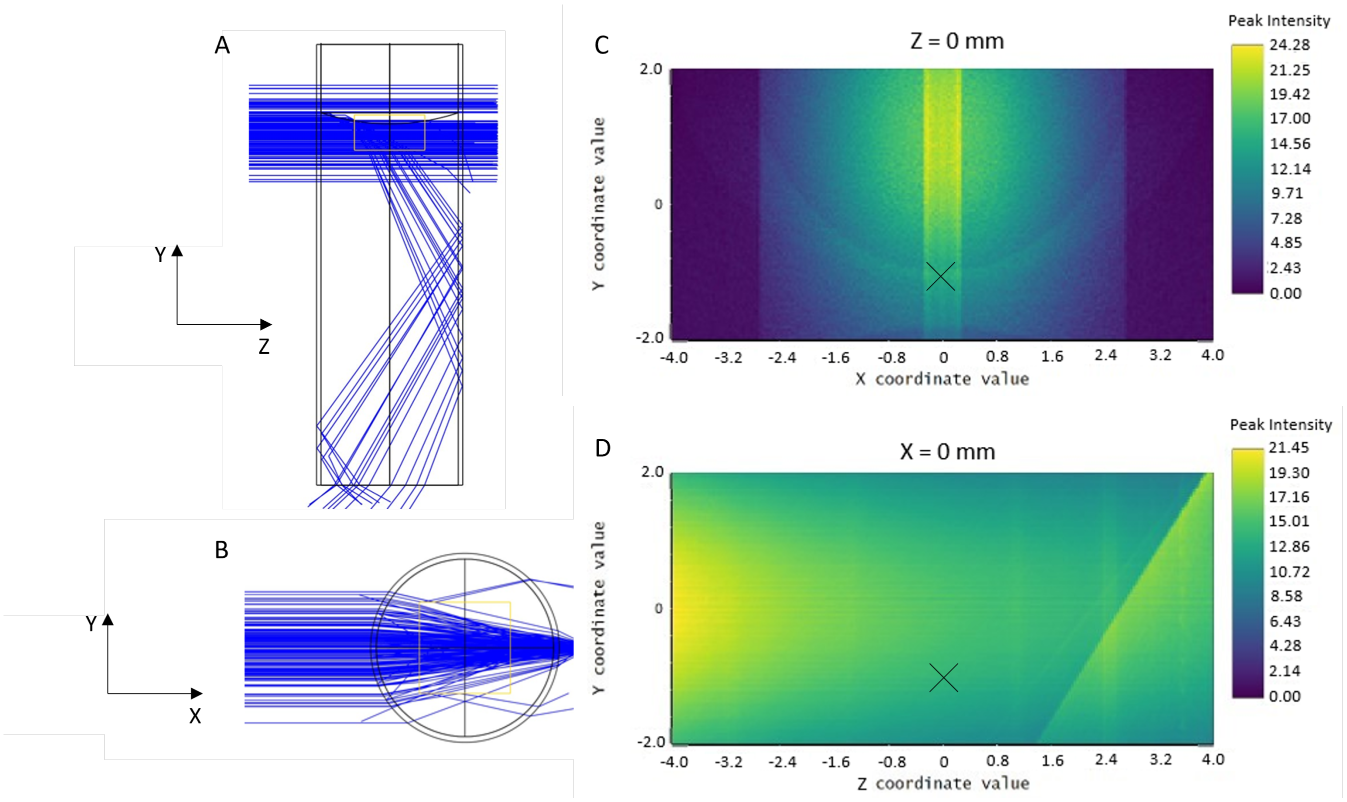

Figure SV: (A) Simulation in Zemax OpticStudio showcasing the behavior of light rays as they encounter total internal reflection when a laser illuminates the meniscus of a solution (side view), (B) Top view, (C) A representation of the peak intensity distribution across the XY plane at a depth of Z = 0 mm within the volume detector, (D) A representation of peak intensity distribution across the YZ plane at a position of X = 0 mm.

temperature. *Crystal Growth and Design* **2012**, *12*, 4554–4561.

(3) https://refractiveindex.info/?shelf=glass&book=SCHOTT-BK&page=N-BK7, [Online; accessed 03-05-2022].

(4) Tan, C.; Huang, Y. Dependence of refractive index on concentration and temperature in electrolyte solution, polar solution, nonpolar solution, and protein solution. *Journal of Chemical & Engineering Data* **2015**, *60*, 2827–2833.
